# *In Vitro* Activity of Solithromycin against Bordetella pertussis, an Emerging Respiratory Pathogen

**DOI:** 10.1128/AAC.01271-16

**Published:** 2016-11-21

**Authors:** Dwight J. Hardy, David Vicino, Prabhavathi Fernandes

**Affiliations:** a Department of Microbiology and Immunology, University of Rochester Medical Center, Rochester, New York, USA; b Cempra, Inc., Chapel Hill, North Carolina, USA

## Abstract

There has been an increase in the number of pertussis cases reported since the introduction of the acellular pertussis vaccine. While children that present with pertussis have a characteristic whooping cough, adults can simply have a persistent, nonspecific cough and remain undiagnosed. Macrolide antibiotics, such as azithromycin, are the currently recommended treatment for pertussis. Solithromycin is a new macrolide and the first fluoroketolide with broad activity against a wide spectrum of bacterial pathogens and has completed clinical development for community-acquired bacterial pneumonia. This study reports the potent *in vitro* activity of solithromycin against a collection of recent isolates of Bordetella pertussis.

## INTRODUCTION

The pertussis component of the DTP vaccine that contained Bordetella pertussis endotoxin in addition to detoxified diphtheria and tetanus toxin was replaced in the 1990s with an acellular pertussis antigen (1). Since the introduction of this vaccine, pertussis outbreaks have been reported (1). In addition, pertactin-deficient strains have been reported to cause outbreaks, pertactin being the immunogenic antigen in the vaccine (2). This selection of pertactin-deficient strains is not unlike the selection of nonvaccine serotypes of pneumococcus in bacterial pneumonia (2) in that the bacterium is selected to escape the protecting antibodies. In infants and young children, the characteristic whoop that ends a pertussis cough is easy to diagnose, but in adults the cough may simply be a persistent cough with various degrees of severity. Since coughs can be caused by multiple respiratory pathogens, including viruses, they are frequently untreated. B. pertussis is difficult to isolate in culture because it is slow growing and can be overgrown by respiratory flora. More recently, nucleic acid amplification assays, including PCR-based assays, have been developed to aid in rapid diagnosis of pertussis (3).

Macrolide antibiotics, especially azithromycin, are frequently prescribed for respiratory tract infections and are active against B. pertussis. Azithromycin remains one of the recommended drugs for the treatment and prophylaxis of pertussis, but an alternate drug is needed. This is because resistance to azithromycin among other respiratory pathogens, such as Streptococcus pneumoniae (4, 5), Streptococcus pyogenes (6) and Mycoplasma pneumoniae (7), has now been reported, and thus azithromycin cannot be used in monotherapy when these antibiotic-resistant pathogens are suspected. In addition, in 2013, the U.S. Food and Drug Administration (FDA) issued a warning that azithromycin can cause a potentially fatal irregular heart rhythm in some patients (8). Therefore, a new macrolide is needed to treat respiratory tract infections. We describe here the *in vitro* activity of solithromycin, a “fourth-generation” macrolide and the first fluoroketolide, which has successfully completed two global phase 3 CABP (community-acquired bacterial pneumonia) trials (9, 10).

(Part of this research was presented at the 53rd Interscience Conference on Antimicrobial Agents and Chemotherapy, Denver, CO, 10 to 13 September 2013 [11].)

## MATERIALS AND METHODS

### Antibacterial agents.

The following drugs were tested: amoxicillin-clavulanate (Sigma, lot 100M08228v; USP, lot J0G109), azithromycin (USP, lot G; Sigma, lot E446421/1v), cefdinir (Sigma, lot 117K1392), cefpodoxime (USP, lot HDKD09), clarithromycin (USP, lot GIG324), doxycycline (Sigma, lot BCBF9827V), penicillin (Sigma, lot BCBF3866V), solithromycin (Cempra, lot EKS11646), and trimethoprim-sulfamethoxazole (Sigma, lots 000M4110V and BCBF0534V). The drugs were dissolved and diluted for testing in accordance with the recommendations of Clinical and Laboratory Standards Institute (CLSI) document M100-S25 (12).

### Organisms.

Strains of B. pertussis were cultured from patient specimens submitted to the clinical laboratories at the University of Rochester Medical Center, Rochester, NY. The MICs of solithromycin (CEM-101) and comparator drugs were determined for 34 clinical strains cultured from nasopharyngeal specimens collected from 2010 to 2014.

### MIC determinations.

Prior to testing, B. pertussis was subcultured onto Mueller-Hinton agar (MHA) supplemented with 5% sheep blood for 48 h at 36°C in ambient air supplemented with 5% CO_2_. The MICs of solithromycin and comparator drugs were determined by agar dilution methodology as described by CLSI document M7-A9 (13) and in Mueller-Hinton agar supplemented with 5% horse blood as recommended by Hoppe and Tschirner (14). Organism suspensions harvested from 48-h cultures were adjusted to yield a final test inoculum of 10^4^ CFU/spot. Inoculated agar plates were incubated for 72 h at 36°C in ambient air supplemented with 5% CO_2_. The MIC endpoints for drugs were read as the concentrations at which no growth, or a significant reduction in growth, was observed by visual inspection after incubation. The performance of test reagents (including drug potency) and equipment and of test personnel was monitored using quality control organisms as recommended by the CLSI. The MICs of all drugs for quality control organisms tested in parallel with test organisms were within acceptable ranges, as recommended by the CLSI (12).

## RESULTS

MICs of solithromycin and comparator drugs for 34 clinical strains of B. pertussis were determined in MHA with 5% sheep blood and MHA with 5% horse blood. The MIC ranges, the 50% MICs, and the 90% MICs of all drugs for strains in both media are presented in Table 1. The MICs of solithromycin were the lowest of the nine drugs tested. Solithromycin was 2- to 4-fold more active than azithromycin and clarithromycin against these strains in both media; solithromycin was also more active than doxycycline and trimethoprim-sulfamethoxazole.

**TABLE 1:** MICs for Bordetella pertussis

Drug	Antimicrobial agent MIC (μg/ml) for B. pertussis (*n* = 34)					
	MHA with 5% sheep blood			MHA with 5% horse blood		
	Range	50%	90%	Range	50%	90%
Solithromycin	0.008–0.03	0.015	0.015	0.004–0.06	0.03	0.03
Azithromycin	0.008–0.06	0.03	0.06	0.015–0.06	0.03	0.06
Clarithromycin	0.015–0.06	0.03	0.06	0.015–0.12	0.12	0.12
Penicillin	0.5–2	1	2	0.5–2	1	1
Amoxicillin-clavulanate	1–2	1	2	2–4	4	4
Cefdinir	2–>32	32	32	8–32	16	32
Cefpodoxime	32–>32	>32	>32	16–>64	64	>64
Doxycycline	0.015–0.12	0.06	0.12	0.03–0.25	0.12	0.25
Trimethoprim-sulfamethoxazole	0.06/1.2–0.12/2.4	0.06/1.2	0.12/2.4	0.25/4.8	0.25/4.8	0.25/4.8

The differences in doubling dilutions between MICs determined in MHA with 5% sheep blood and in MHA with 5% horse blood are shown in Table 2. The MICs of solithromycin, azithromycin, clarithromycin, doxycycline, trimethoprim-sulfamethoxazole, and amoxicillin-clavulanate were generally lower in media with sheep blood than the MICs determined in media with horse blood; these differences, however, do not suggest interpretive differences in the MICs between the two media.

**TABLE 2:** Differences in doubling dilutions between MICs determined in MHA with sheep blood versus MICs determined in MHA with horse blood

Drug	Difference in doubling dilution^*a*^						
	−2	−1	0	1	2	3	4
Solithromycin	5	2	1	20	4	1	1
Azithromycin		8	11	10	3	2	
Clarithromycin		2	6	11	15		
Penicillin		10	17	4	3		
Amoxicillin-clavulanate			1	17	16		
Cefdinir	1	22	6	2		3	
Cefpodoxime		2	24	8			
Doxycycline	1	2	6	7	6	8	3
Trimethoprim-sulfamethoxazole				4	30		

a The values in the body of the table are the number of B. pertussis strains with indicated difference in doubling dilutions between MICs determined in the different media.

## DISCUSSION

There has been increased surveillance and reporting of pertussis to public health departments in recent years due to increased recognition of pertussis among clinicians, greater use of laboratory diagnostic tests (especially nucleic acid amplification-based methods), and waning immunity from vaccines. Prior to the introduction of the pertussis vaccine in the 1940s, more than 200,000 cases of pertussis were reported annually and, in the last century, pertussis was one of the most common childhood diseases and a major cause of childhood mortality in the United States (1). The availability of the vaccine resulted in decreased incidence by more than 80% compared to the prevaccine era. Since the 1990s, however, there has been an increase in the number of reported cases of pertussis, and in 2010 a total of 27,500 cases were reported in the United States (1).

Since B. pertussis is difficult to culture and testing for B. pertussis by any method must be specifically requested, therapy for severe respiratory illness is empirical. Solithromycin has been shown to be active against a wide variety of respiratory pathogens, including azithromycin-resistant pneumococcus (5). Unlike older macrolides, solithromycin does not prolong the cardiac QT interval (15). The MICs of solithromycin for 100% of the 34 clinical strains of B. pertussis in this study were 0.03 μg/ml in media with sheep blood and 0.06 μg/ml in media with horse blood, and solithromycin was the most potent of the nine antibiotics tested. Solithromycin was 2- to 4-fold more active than azithromycin and clarithromycin against these strains. Solithromycin is active against a broad spectrum of respiratory pathogens and has been successfully tested in monotherapy for moderate to moderately severe CABP in two phase 3 clinical trials. The results from the present study show that solithromycin could provide coverage against pertussis when treating severe respiratory tract infections.
